# Supplementary material for: Aromatized liposomes for sustained drug delivery
Source: Nat Commun. 2023 Oct 20;14:6659. doi: 10.1038/s41467-023-41946-8 (PMC10589217; doi:10.1038/s41467-023-41946-8)
Supplement: Supplementary file 1 — Supplementory Information [file 41467_2023_41946_MOESM1_ESM.pdf]

Supplementary Information

Aromatized Liposomes for Sustained Drug Delivery

Yang Li[1], Tianjiao Ji[1], Matthew Torre[2], Rachelle Shao[1], Yueqin Zheng[1], Dali Wang[1], Xiyu Li[1], Andong Liu[1], Wei Zhang[1], Xiaoran Deng[1], Ran Yan[1], and Daniel S. Kohane[1]*

[1]Laboratory for Biomaterials and Drug Delivery, Department of Anesthesiology, Division of Critical Care Medicine, Children's Hospital Boston, Harvard Medical School, Boston, MA 02115, United States
[2]Department of Pathology, Brigham and Women's Hospital, Boston, MA 02115, United States
* To whom correspondence may be addressed.
E-mail: daniel.kohane@childrens.harvard.edu (D.S. Kohane). Tel: 1-617-919-2364.

Supplementary Methods

Synthesis of aromatic group-modified phospholipids. 16-bromohexadecanoic acid (1.34 g, 4.00 mmol), acetyl chloride (0.79 g, 10.00 mmol) and anhydrous methanol (20 mL) were placed in a 100 mL round-bottomed flask. Acetyl chloride is flammable and corrosive. 16-bromohexadecanoic acid may cause irritation. The solution was stirred overnight at room temperature. The solvent was removed under reduced pressure and the resulting solids were resuspended in dichloromethane, then washed with $NaHCO_3$ (2 x 200 mL) and brine (2 x 200 mL). The organic phase was separated, dried over Na_2SO_4. The solvent was removed under reduced pressure to yield methyl 16-bromohexadecanoate in excellent yield (90-95%). ^1H NMR ($CDCl_3$, 400MHz, ppm, δ): 3.66 (s, 3H), 3.44-3.36 (t, 2H), 2.33-2.25 (t, 2H), 1.89-1.79 (m, 2H), 1.66-1.55 (m, 2H), 1.47-1.36 (m, 2H), 1.34-1.21 (m, 20H). ^{13}C NMR ($CDCl_3$, 400MHz, ppm, δ): 174.09, 51.33, 34.06, 33.78, 32.86, 29.63, 29.61, 29.58, 29.54, 29.45, 29.26, 29.15, 28.78, 28.19, 24.95. ESI-MS: m/z calculated for $C_{17}H_{33}BrO_2$ $[M+H]^+$: 349.2; observed: 349.2.

The methyl 16-bromohexadecanoate (1.40 g, 4.00 mmol) and K_2CO_3 (1.38 g, 10.00 mmol) were then added to 30 mL MeCN in a 100 mL round-bottomed flask and heated to 60 ^{o}C. Phenol (0.47 g, 5.00 mmol) was dissolved in 1 mL MeCN and added to the reaction mixture. Phenol may cause irritation. The reaction mixture was stirred for 16 hours at 60 ^{o}C. The reaction mixture was cooled to room temperature, filtered to remove the salt. The solvent was removed under reduced pressure and the obtained material was resuspended in dichloromethane, then washed with $NaHCO_3$ (2 x 200 mL) and brine (2 x 200 mL). The organic phase was separated, dried over Na_2SO_4. The solvent was removed under reduced pressure to give a crude residue. The obtained crude material was purified by silica gel flash chromatography eluting with 10:1 hexanes/ethyl acetate to yield methyl 16-phenoxyhexadecanoate in good yield (69-75%). ^1H NMR ($CDCl_3$, 400MHz, ppm, δ): 7.34-7.22 (m, 2H), 6.96-6.85 (m, 3H), 3.99-3.91 (t, 2H), 3.66 (s, 3H), 2.35-2.25 (t, 2H), 1.83-1.71 (m, 2H), 1.68-1.56 (m, 2H), 1.50-1.39 (m, 2H), 1.39-1.20 (m, 20H). ^{13}C NMR ($CDCl_3$, 400MHz, ppm, δ): 174.11, 159.28, 129.52, 120.58, 114.66, 68.05, 51.34, 34.21, 29.78, 29.77, 29.72, 29.57, 29.55, 29.45, 29.38, 29.20, 26.21, 24.82. ESI-MS: m/z calculated for $C_{23}H_{38}O_3$ $[M+H]^+$: 363.3; observed: 363.3.

Methyl 16-phenoxyhexadecanoate (1.45 g, 4.00 mmol), tetrahydrofuran (20 mL) and deionized water (20 mL) were placed in a 100 mL round-bottomed flask. 1 M NaOH (20.00 mmol) was then added dropwise, and the reaction mixture was stirred for 16 hours at room temperature. Tetrahydrofuran is flammable and may cause irritation. The reaction mixture was diluted with ethyl acetate (100 mL), washed with 10% HCl (50 mL) then with deionized water (2 x 100 mL) and brine (100 mL). The organic phase was separated, dried over Na_2SO_4. The solvent was removed under reduced pressure to yield 16-phenoxyhexadecanoic acid in good yield (70-80%). ^1H NMR ($CDCl_3$, 400MHz, ppm, δ): 7.34-7.22 (m, 2H), 6.96-6.85 (m, 3H), 3.99-3.91 (t, 2H), 2.39-2.30 (t, 2H), 1.84-1.72 (m, 2H), 1.70-1.58 (m, 2H), 1.52-1.41 (m, 2H), 1.39-1.20 (m, 20H). ^{13}C NMR ($CDCl_3$, 400MHz, ppm, δ): 180.31, 159.28, 129.52, 120.58, 114.66, 68.05, 34.21, 29.78, 29.77, 29.72, 29.57, 29.55, 29.45, 29.38, 29.20, 26.21, 24.82. ESI-MS: m/z calculated for $C_{17}H_{33}BrO_2$ $[M+H]^+$: 349.3; observed: 349.3.

16-phenoxyhexadecanoic acid (0.35 g, 1.00 mmol), 16:0 lyso-PC (0.44 g, 0.90 mmol) and 1-methylimidazole (0.25 g, 3.00 mmol) and 2,6-dichlorobenzoyl chloride (0.42 g, 2.00 mmol) were combined in $CHCl_3$ (6 mL) in a 100 mL round-bottomed flask resulting mixture was stirred for 16 h at room temperature. 1-methylimidazole is an acute toxic health hazard. 1-methylimidazole and 2,6-dichlorobenzoyl chloride are corrosive. The reaction mixture was then concentrated by removing the solvent under reduced pressure to give a crude residue. The resulting crude was purified by silica gel chromatography (1:1 MeOH/CH_2Cl_2) yielding 1-palmitoyl-2-(16-phenoxy) palmitoyl-sn-glycero-3-phosphocholine (Ph-DPPC) as a white wax in moderate yield (50-60%). ^{1}H NMR (CD_3OD, 400MHz, ppm, δ): 7.28-7.20 (m, 2H), 6.94-6.85 (m, 3H), 5.29-5.20 (m, 1H), 4.48-4.39 (m, 1H), 4.32-4.22 (m, 2H), 4.21-4.13 (m, 1H), 4.04-3.98 (t, 2H), 3.97-3.92 (t, 2H), 3.66-3.61 (t, 2H), 3.22 (s, 9H), 2.38-2.28 (m, 4H), 1.80-1.72 (m, 2H), 1.66-1.55 (m, 4H), 1.52-1.42 (m, 4H), 1.41-1.22 (m, 44H) 0.93-0.86 (t, 3H). ^{13}C NMR ($CDCl_3$, 400MHz, ppm, δ): 174.93, 173.62, 160.61, 130.39, 121.49, 115.53, 71.79, 68.88, 67.50, 64.92, 63.69, 60.49, 54.75, 35.12, 34.94, 33.08, 30.81, 30.78, 30.71, 30.66, 30.52, 30.49, 30.46, 30.45, 30.23, 30.20, 27.19, 26.05, 26.03, 23.74.. ESI-MS: m/z calculated for $C_{46}H_{84}NO_9P$ $[M+H]^+$: 826.6; observed: 826.6.

The yield of the above coupling reaction was extremely low with commonly used coupling reagents such as N,N′-dicyclohexylcarbodiimide (DCC), N,N′-diisopropylcarbodiimide (DIC), and 1-ethyl-3-(3-dimethylaminopropyl) carbodiimide (EDC), probably due to the poor solubility of lysoPC in organic solvents like chloroform. 2,6-dichlorobezoyl chloride and 1-methylimidazole was selected as the coupling reagent systems for the synthesis of acyl chain-modified phospholipids due to their high potency in activating fatty acids. The coupling reaction was completed within 12 hours.

Supplementary Figures

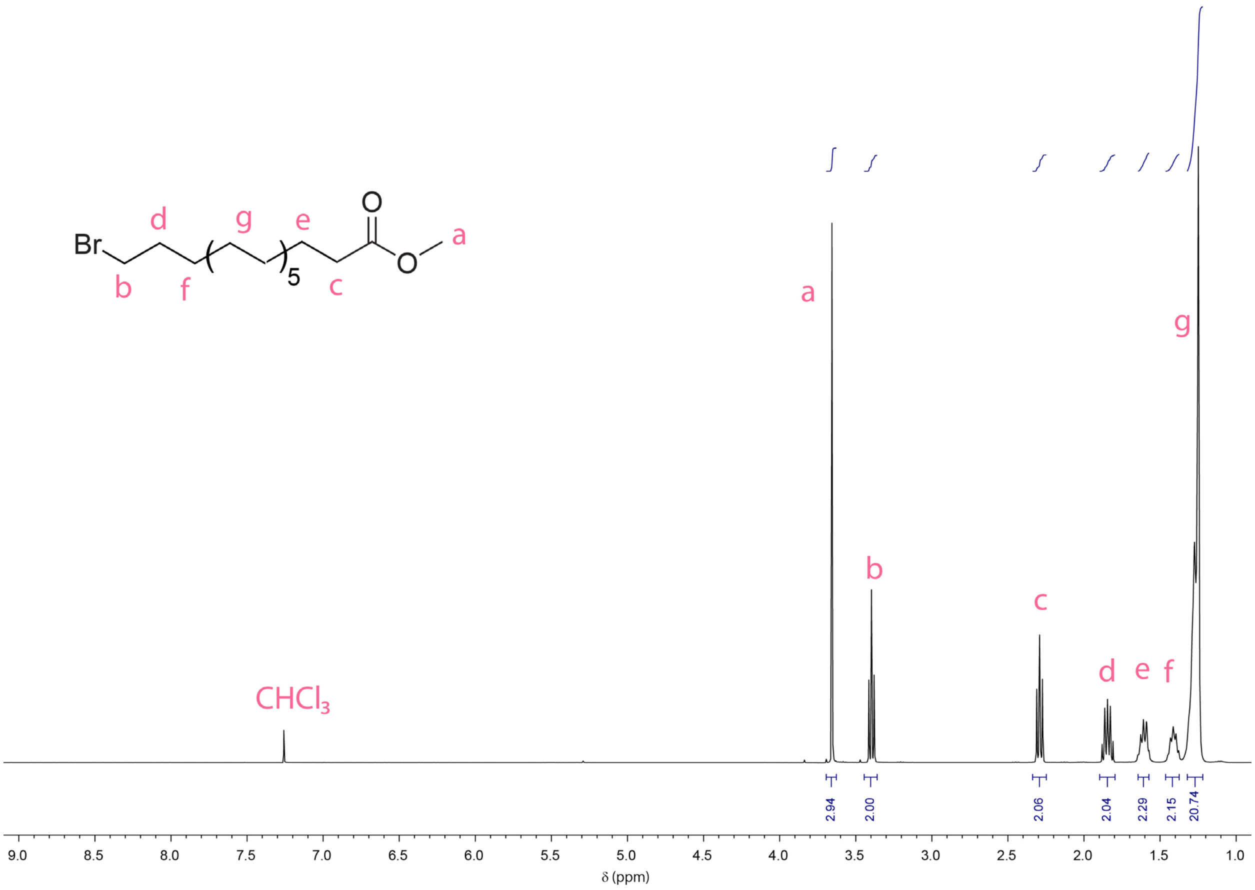

Supplementary Figure 1. ^{1}H-NMR spectrum of methyl 16-bromohexadecanoate.

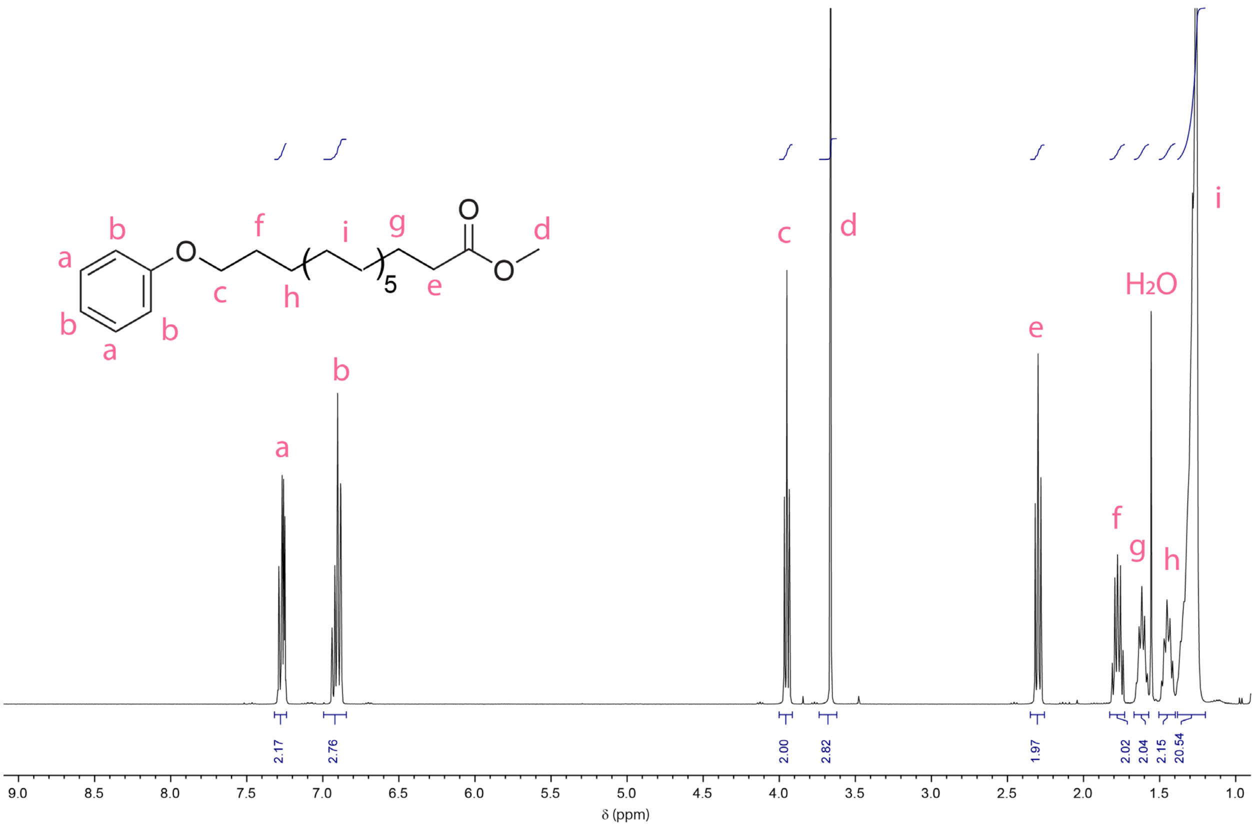

Supplementary Figure 2. ^{1}H-NMR spectrum of methyl 16-phenoxyhexadecanoate.

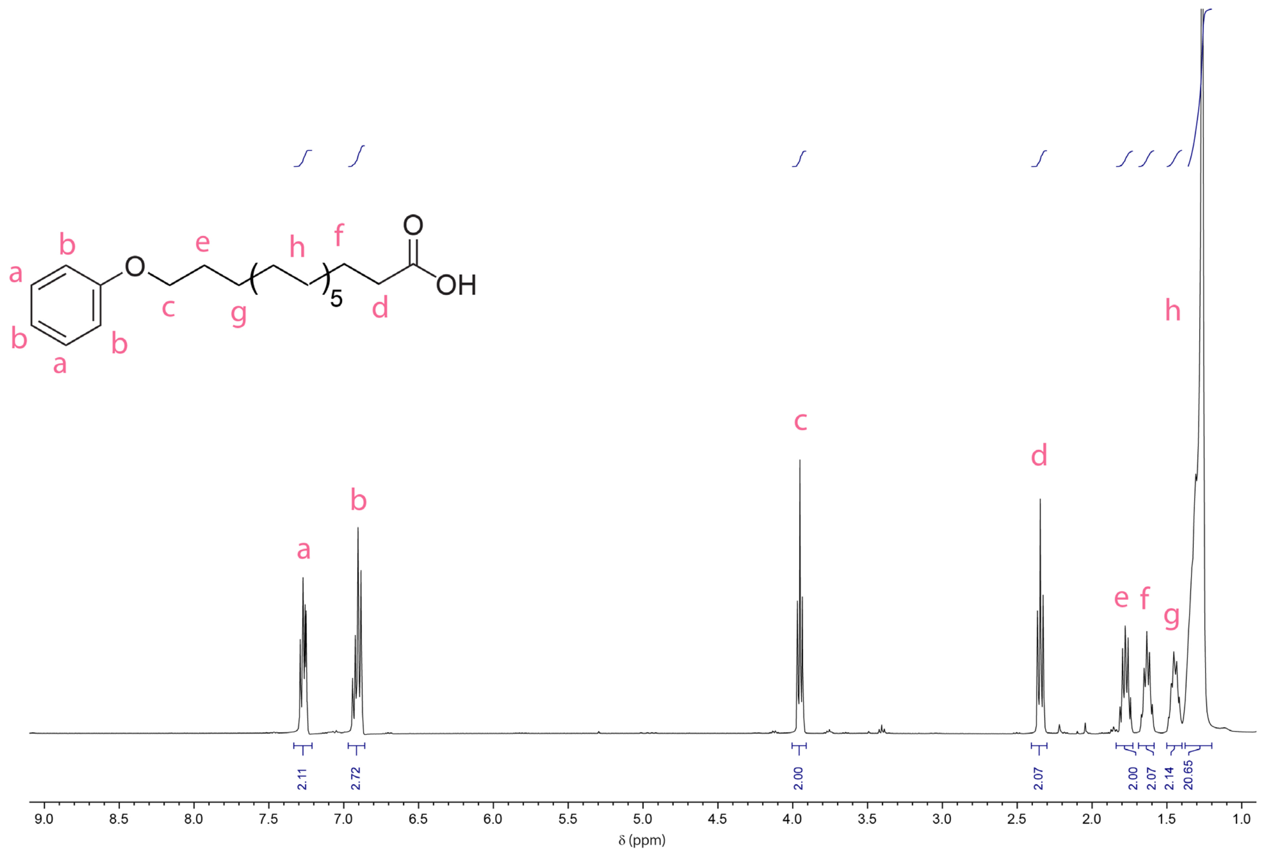

Supplementary Figure 3. ^{1}H-NMR spectrum of 16-phenoxyhexadecanoic acid.

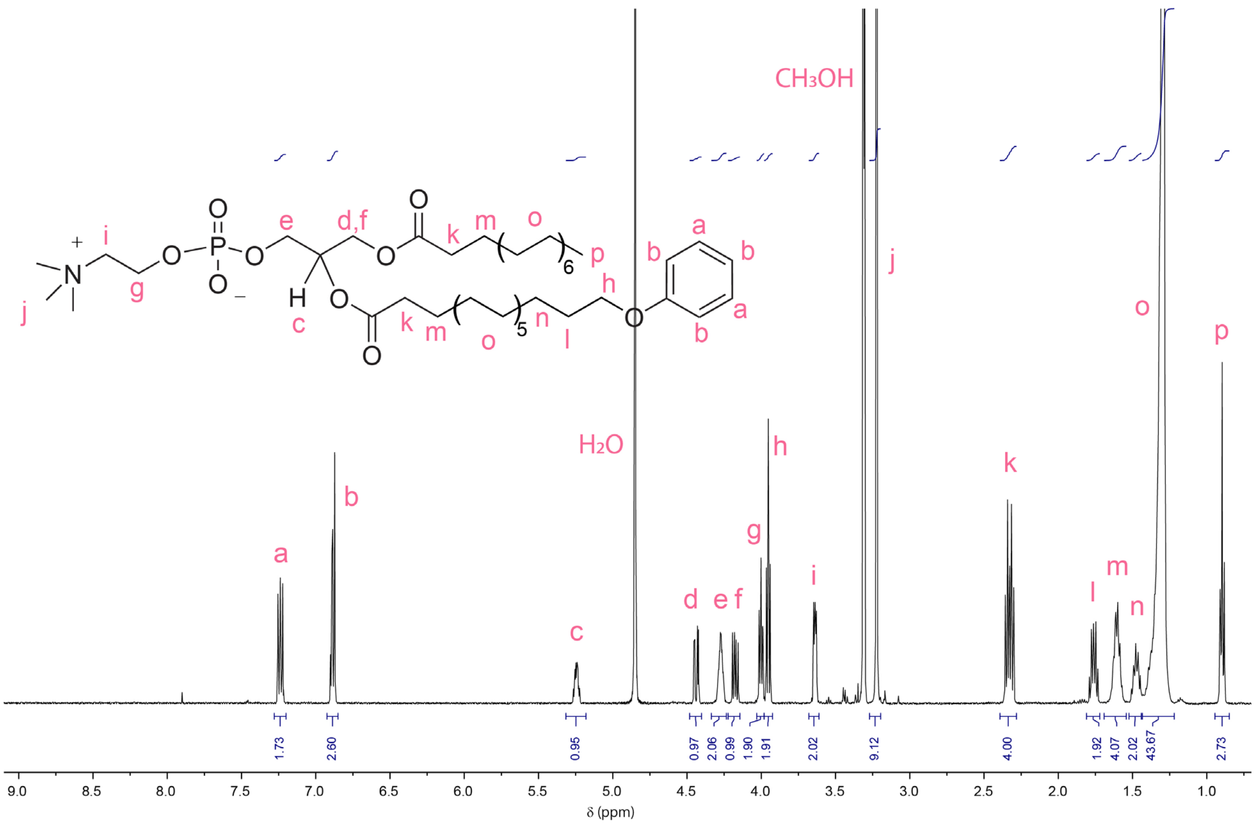

Supplementary Figure 4. ^{1}H-NMR spectrum of 1-palmitoyl-2-(16-phenoxy)palmitoyl-sn-glycero-3-phosphocholine (Ph-DPPC). Peak *a, b, h* and *l* were new peaks resulting from the conjugation of the phenoxy group.

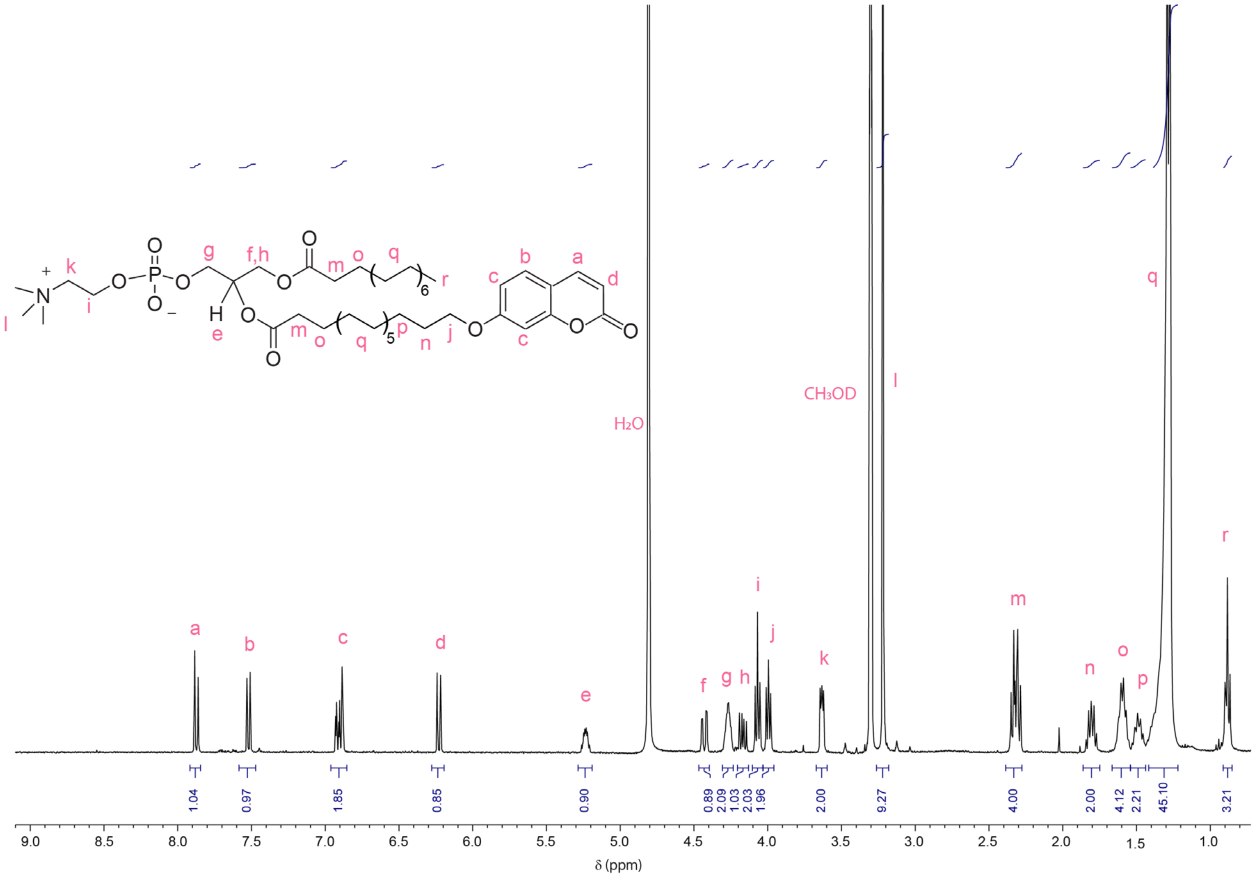

Supplementary Figure 5. ^{1}H-NMR spectrum of 1-palmitoyl-2-(16-coumarin)palmitoyl-sn-glycero-3-phosphocholine (CM-DPPC). Peak *a, b, c, d, h* and *l* were new peaks resulting from the conjugation of the coumarin group.

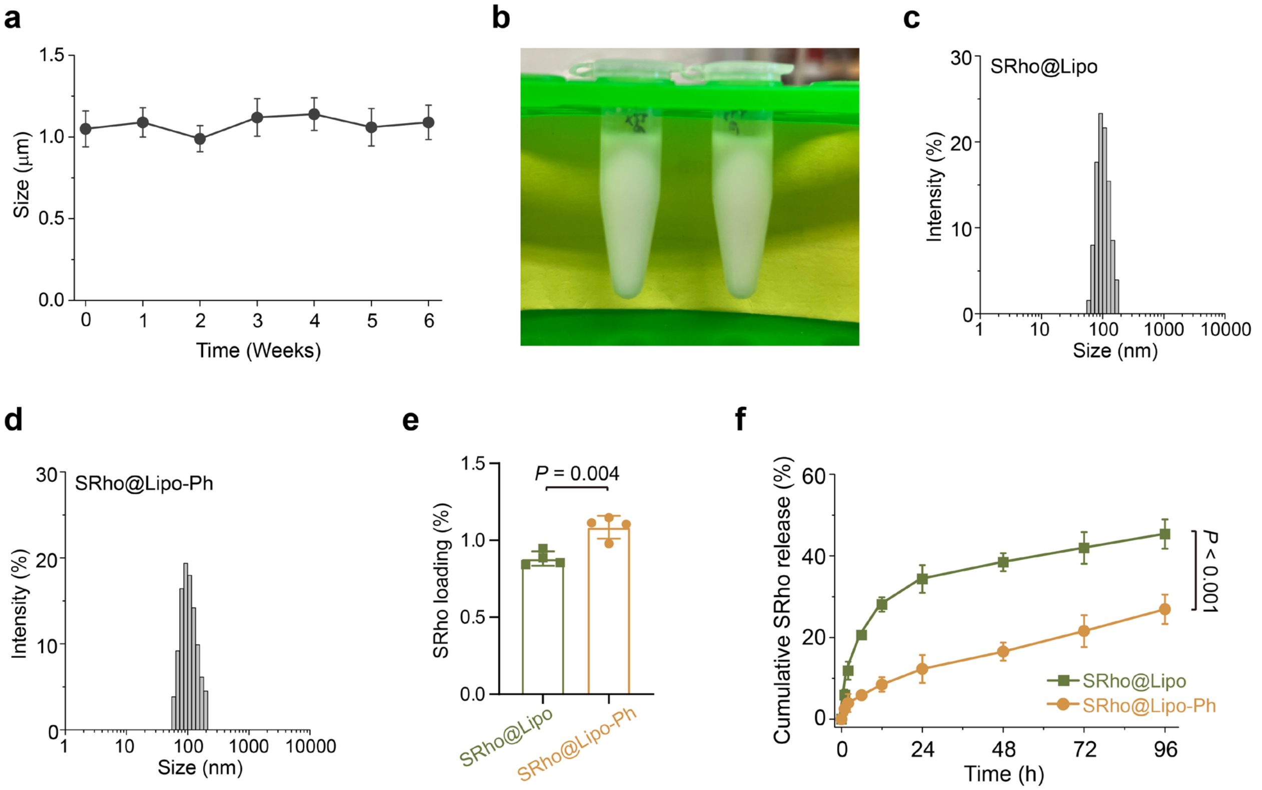

Supplementary Figure 6. Characterizations of aromatized liposomes. a, The stability of aromatized liposome. Data are presented as mean ± SD. **b,** Solution of aromatized liposomes. **c,** Size of nanoscale liposomes encapsulating Sulforhodamine B (SRho@Lipo) after extrusion. **d**, Size of nanoscale aromatized liposomes encapsulating Sulforhodamine B (SRho@Lipo-Ph) after extrusion. **e**, SRho loading in different nanoscale liposomes. Data are presented as mean ± SD. Statistical analysis was performed using the two-tailed t-test. $P = 0.0035$. **f**, Cumulative release of SRho from different formulations at 37 °C. In **e-f**, green square represents SRho@Lipo and orange circle represents SRho@Lipo-Ph. Data are presented as mean ± SD, n = 4 independent experiments. Statistical analysis was performed using the two-tailed t-test. The p-values compares groups at 96 h. P < 0.0001. *$P < 0.05$, **$P < 0.01$. ***$P < 0.001$.

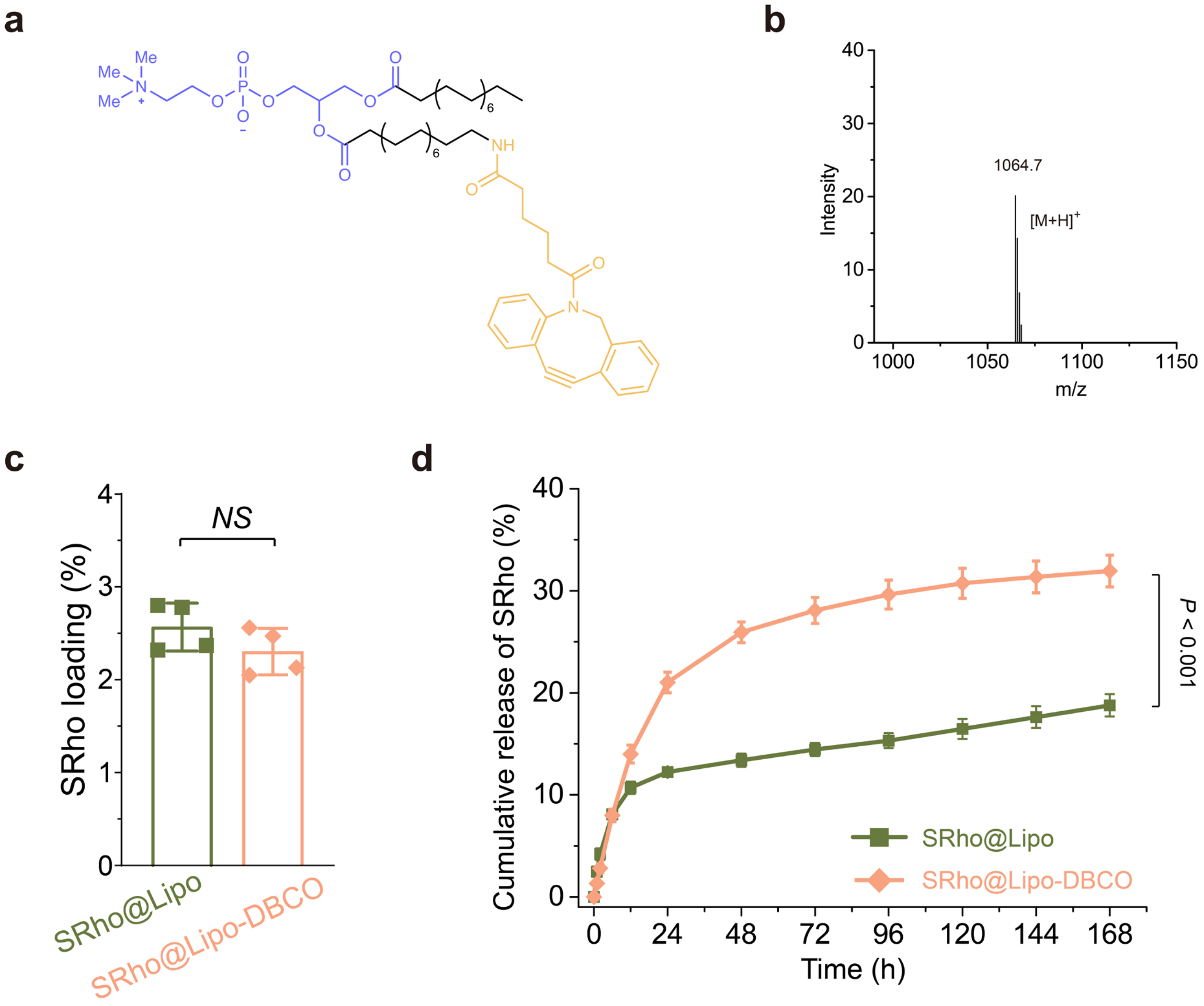

Supplementary Figure 7. **Dibenzocyclooctyne-modified liposomes (Lipo-DBCO). a**, Structure of DBCO-modified phospholipids (DBCO-DPPC). The DBCO group was covalently conjugated to the acyl chain of phospholipid via polar amide bonds (labeled in orange). **b**, Mass spectra of DBCO-DPPC. [M] represents the mass calculated (m/z) and $[M+H]^+$ represent mass found in LCMS measurement. **c,** SRho loading in DBCO-modified liposomes (SRho@Lipo-DBCO, orange diamond) and unmodified liposome (SRho@Lipo, green square). Data are presented as mean ± SD, n =4 independent experiments. Statistical analysis was performed using the unpaired two-tailed t-test. P = 0.1907. **d**, Cumulative release of SRho from SRho@Lipo-DBCO. Data are presented as mean ± SD, n =4 independent experiments. Statistical analysis was performed using the two-tailed t-test. $P < 0.0001$ compares groups at 168 h. *$P < 0.05$, **$P < 0.01$. ***$P < 0.001$.

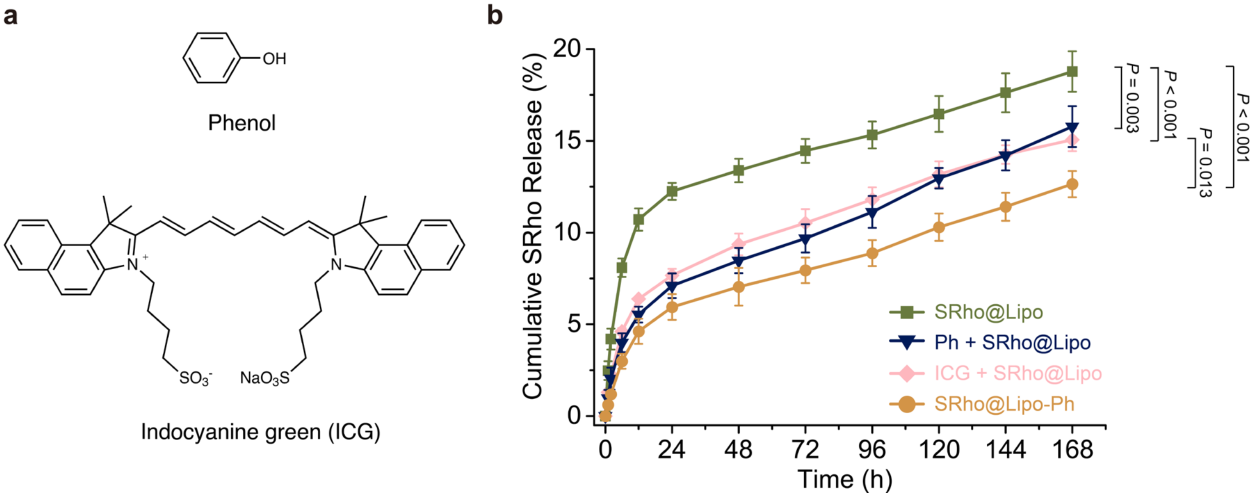

Supplementary Figure 8. Release kinetics of Sulforhodamine B (SRho) from liposomes containing physically encapsulated aromatic molecules or covalently conjugated aromatic groups. a, Chemical structures of phenol and indocyanine green (ICG). **b,** Cumulative release of Sulforhodamine B (SRho) from different formulations**.** Ph+SRho@Lipo and ICG+SRho@Lipo are liposomes containing physically encapsulated phenol (Ph) or indocyanine green (ICG). SRho@Lipo-Ph are liposomes containing covalently conjugated phenoxy group. Green square, navy triangle, pink diamond, and orange circle represent liposomes encapsulating SRho, liposomes encapsulating Ph and SRho, liposomes encapsulating ICG and SRho, aromatized liposomes encapsulating SRho, respectively. Data are presented as mean ± SD, n =4 independent experiments. Statistical analysis was performed using one-way ANOVA with a Tukey post hoc test; p-values compare groups at 168 h. SRho@Lipo vs Ph+SRho@Lipo, $P = 0.0028$. SRho@Lipo vs ICG+SRho@Lipo, $P = 0.0005$. SRho@Lipo vs SRho@Lipo-Ph, $P < 0.0001$. ICG+SRho@Lipo vs SRho@Lipo-Ph, $P = 0.0133$. *$P < 0.05$, **$P < 0.01$. ***$P < 0.001$.

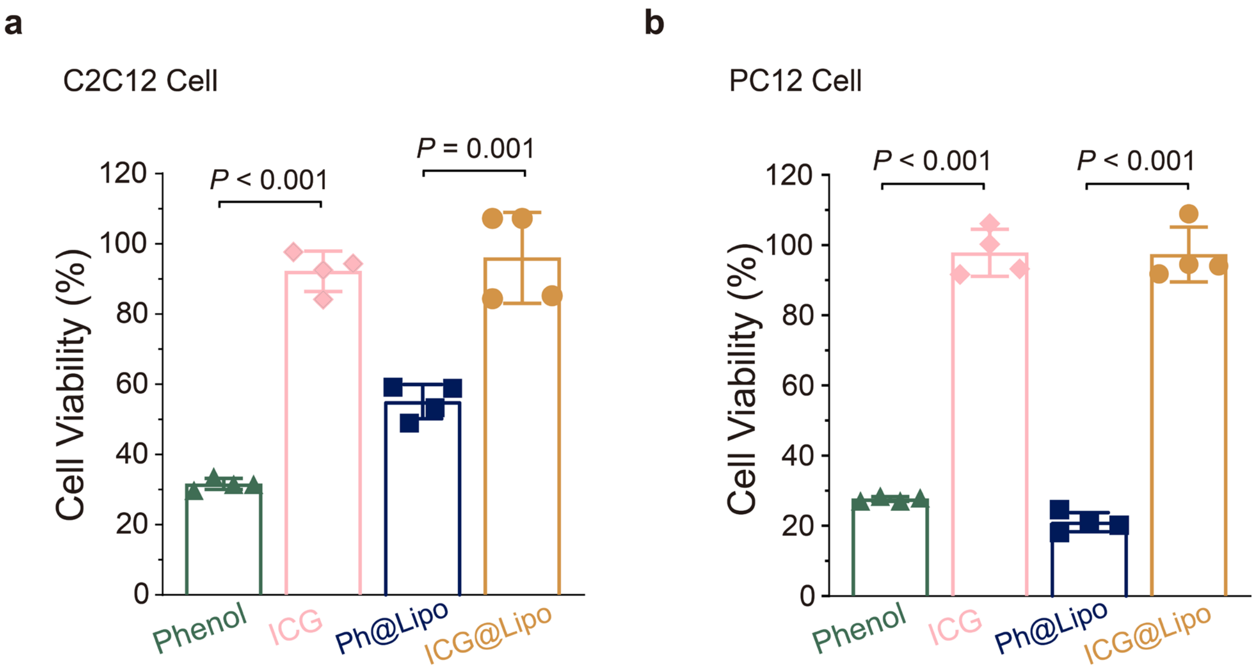

Supplementary Figure 9. **Cytotoxicity of aromatic molecules. a**, Cell viability of C2C12 cells incubated with free aromatic molecules and liposomes loaded with aromatic molecules physically (@Lipo) or covalently (-Lipo). The concentration of Phenol and Indocyanine Green (ICG) were 5 mM. Data are presented as mean ± SD, n =4 independent experiments. Statistical analysis was performed using the two-tailed t-test. Phenol vs ICG, $P < 0.0001$. Ph@Lipo vs ICG@Lipo, $P = 0.0010$. **b**, Cell viability of PC12 cells incubated with free aromatic molecules and liposomes loaded with aromatic molecules physically (@Lipo) or covalently (-Lipo). The concentration of Phenol and ICG were 5 mM. Data are presented as mean ± SD, n =4 independent experiments. Statistical analysis was performed using the two-tailed t-test. Phenol vs ICG, $P < 0.0001$. Ph@Lipo vs ICG@Lipo, $P < 0.0001$. $P = 0.0133$. *$P < 0.05$, **$P < 0.01$. ***$P < 0.001$. In **a-b**, green triangle, pink diamond, navy square, and orange circle represent Phenol, ICG, liposome encapsulating Phenol, and liposome encapsulating ICG, respectively.

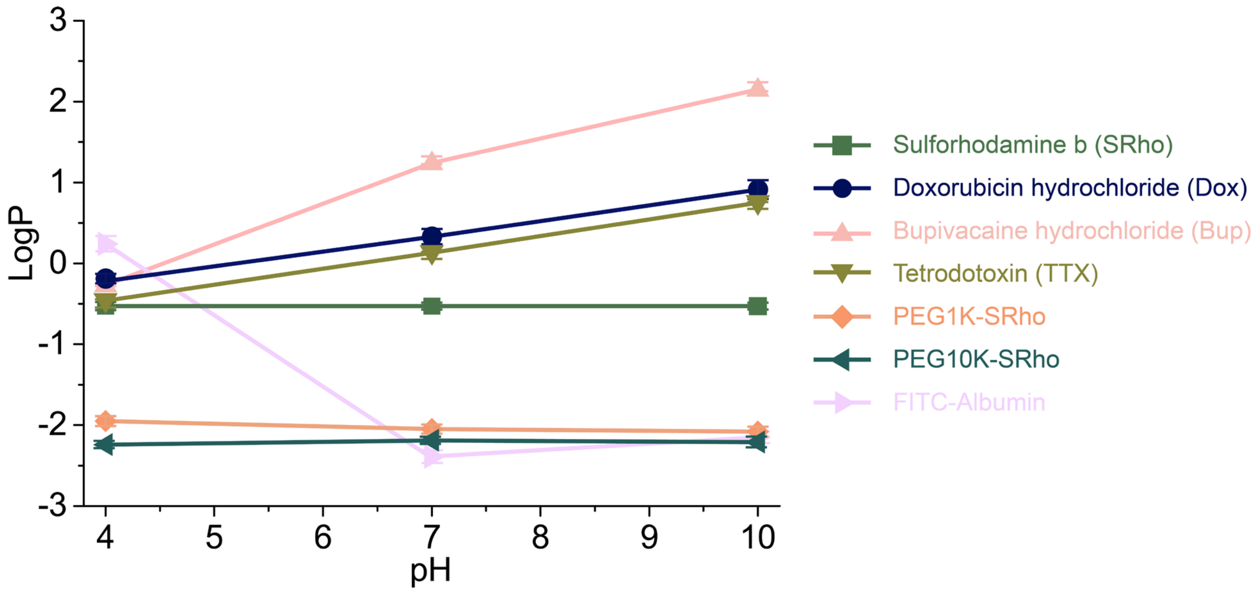

Supplementary Figure 10. pH-dependent partition coefficients of different payloads. Octanol−water partition coefficients were quantified using a miniaturized shake-flask approach (See Partition coefficient quantification section in Methods). Also see the same section for explanation of which logPs were used in our analyses. Square represents Sulforhodamine B (SRho); Circle represents Doxorubicin hydrochloride (Dox); Up-pointing triangle represents Bupivacaine hydrochloride (Bup); Down-pointing triangle represents Tetrodoxtoxin (TTX); Diamond represents SRho-conjugated polyethylene glycol with a molecular weight of 1,000 (PEG1K-SRho); Left-pointing triangle represents SRho-conjugated polyethylene glycol with a molecular weight of 10,000 (PEG10K-SRho); Right-pointing triangle represents albumin-fluorescein isothiocyanate conjugate (FITC-Ab). Data are presented as mean ± SD, n = 4 independent experiments. Statistical analysis was performed using one-way ANOVA with a Tukey post hoc test; p-values compare groups at pH 7.4, the pH at which the compounds were encapsulated. Bup vs Dox, $P < 0.0001$; Dox vs TTX, $P = 0.0020$; TTX vs SRho, $P < 0.0001$; PEG1k-SRho vs PEG10k-SRho, $P = 0.0346$; PEG1k-SRho vs FITC-Ab, $P < 0.0001$; PEG10k-SRho vs FITC-Ab, $P = 0.0057$. *$P < 0.05$, **$P < 0.01$. ***$P < 0.001$.

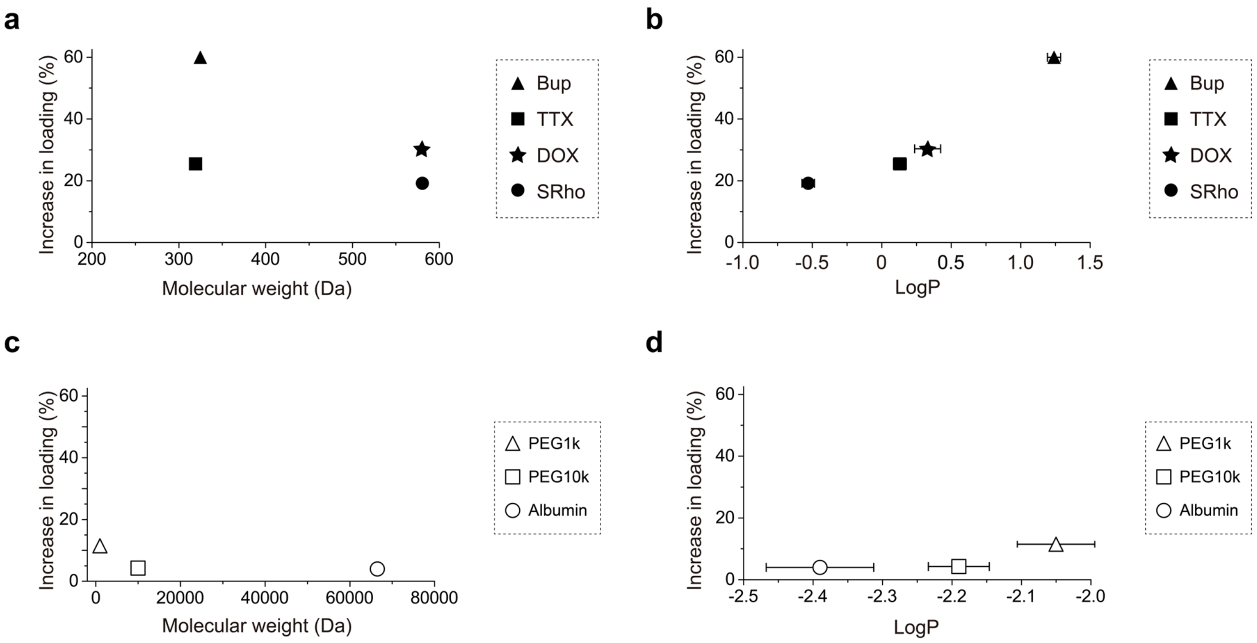

Supplementary Figure 11. Effect of liposome aromatization on the loading of different payloads. a, b, the increase of small molecule loading as a function of molecular weight (a) and hydrophilicity (b). In **b**, logP values are presented as mean ± SD, n = 4 independent experiments. Statistical analysis was performed using one-way ANOVA with a Tukey post hoc test. Bup vs Dox, $P < 0.0001$; Dox vs TTX, $P = 0.0020$; TTX vs SRho, $P < 0.0001$. **c, d**, the increase of macromolecule loading as a function of molecular weight (c) and hydrophilicity (d). In **d**, logP values are presented as mean ± SD, n = 4 independent experiments. Statistical analysis was performed using one-way ANOVA with a Tukey post hoc test. PEG1k-SRho vs PEG10k-SRho, P = 0.0346; PEG1k-SRho vs FITC-Ab, $P < 0.0001$; PEG10k-SRho vs FITC-Ab, $P = 0.0057$. *$P < 0.05$, **$P < 0.01$. ***$P < 0.001$.

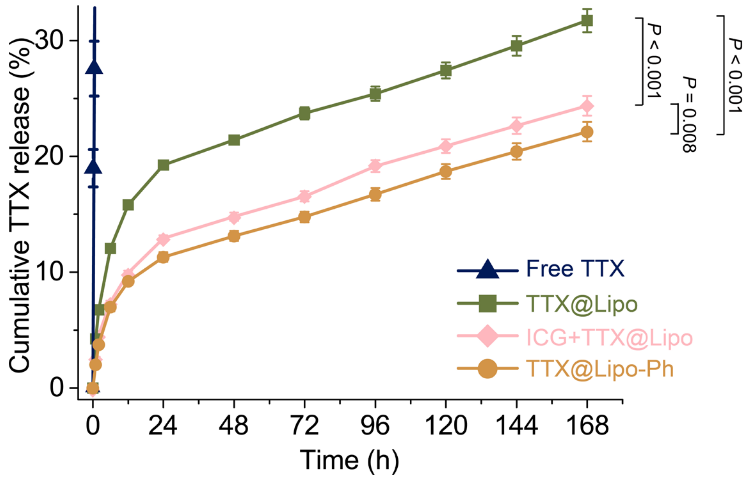

Supplementary Figure 12. Tetrodotoxin (TTX) in liposomes. Cumulative TTX release from different formulations at 37 °C. TTX concentrations were quantified by TTX ELISA. Navy triangle, green square, pink diamond, and orange circle represent free TTX, liposomes encapsulating TTX (TTX@Lipo), liposomes encapsulating indocyanine green (ICG) and TTX (ICG+TTX@Lipo), aromatized liposome encapsulating TTX (TTX@Lipo-Ph), respectively. Data are presented as mean ± SD, n =4 independent experiments. Statistical analysis was performed using one-way ANOVA with a Tukey post hoc test; p-values compare groups at 168 h. TTX@Lipo vs ICG+TTX@Lipo, $P < 0.0001$. TTX@Lipo vs TTX@Lipo-Ph, $P < 0.0001$. ICG+TTX@Lipo vs TTX@Lipo-Ph, $P = 0.0080$. $^{*}P < 0.05$, $^{**}P < 0.01$. $^{***}P < 0.001$.

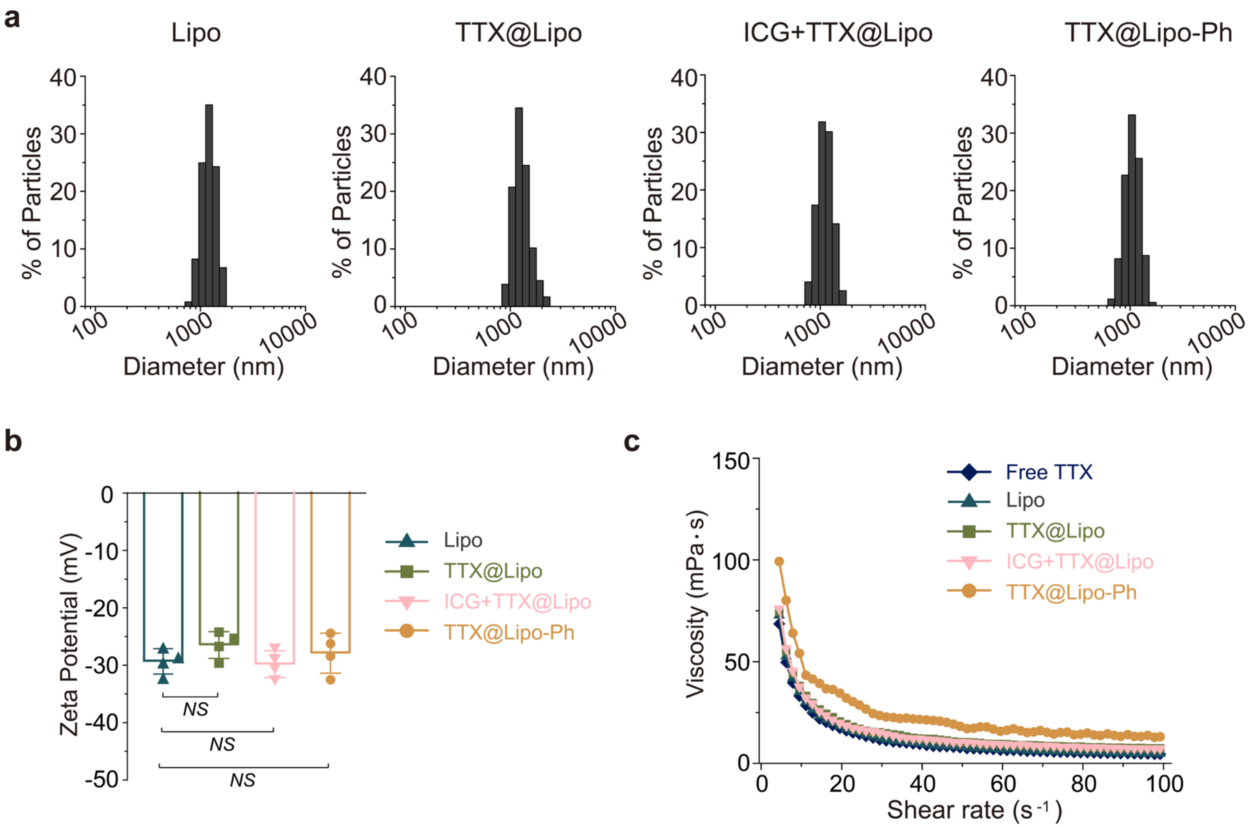

Supplementary Figure 13. Physicochemical characterizations of liposomes encapsulating TTX. a, Volume-weighted diameters of liposomes measured by dynamic light scattering. **b**, Zeta potential of liposomes. Navy diamond, dark green up-pointing triangle, green square, pink down-pointing triangle, and orange circle represent free tetrodotoxin (TTX), blank liposomes (Lipo), liposomes encapsulating TTX (TTX@Lipo), liposomes encapsulating Indocyanine Green (ICG) and TTX (ICG+TTX@Lipo), aromatized liposomes encapsulating TTX (TTX@Lipo-Ph). Data are presented as mean ± SD, n =4 independent experiments. Statistical analysis was performed using one-way ANOVA with a Tukey post hoc test. Lipo vs TTX@Lipo, P = 0.4543. Lipo vs ICG+TTX@Lipo, P = 0.9931. Lipo vs TTX@Lipo-Ph, P = 0.8697. *$P < 0.05$, **$P < 0.01$. ***$P < 0.001$. **c**, Viscosity of liposomal formulations as a function of shear rate.

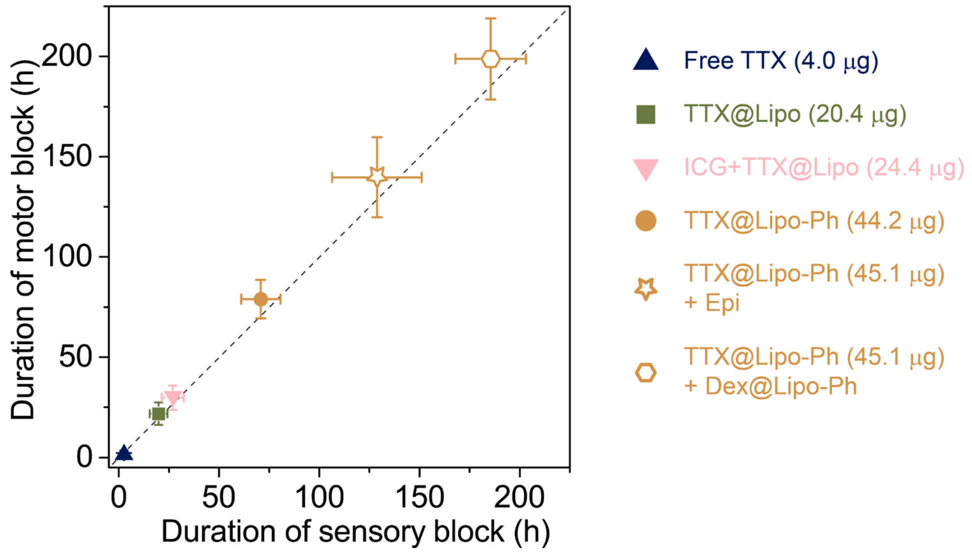

Supplementary Figure 14. Comparison of the durations of sensory and motor blocks from TTX formulations. Data are presented as mean ± SD, n =4 biologically independent animals. The diagonal dotted line denotes equal durations of sensory and motor block.

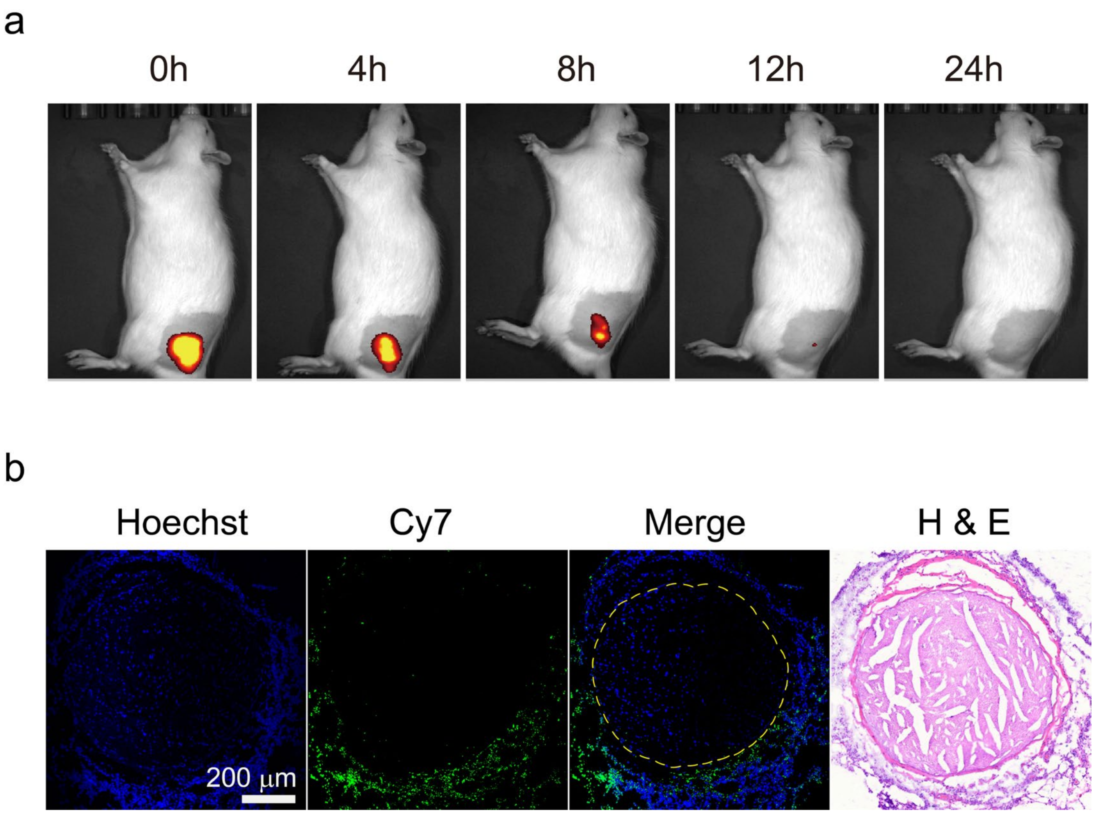

Supplementary Figure 15. Retention and localization of free Cy7. a, Representative fluorescent in vivo imaging of rats injected at the sciatic nerve with free Cy7. Fluorescence intensity is represented as radiant efficiency. The experiment was repeated in 4 biologically independent animals, yielding similar results. **b**, Representative fluorescent confocal photomicrographs 0.5 h after administration of free Cy7, with corresponding hematoxylin-eosin-stained (H&E) sections. Scale bar: 200 μm. Blue: Hoechst 33342, indicating cell nuclei; Green: Cy7, indicating dye-labeled liposomes. The yellow dotted line indicates the nerve perimeter. The experiment was repeated in 4 biologically independent animals, yielding similar results.

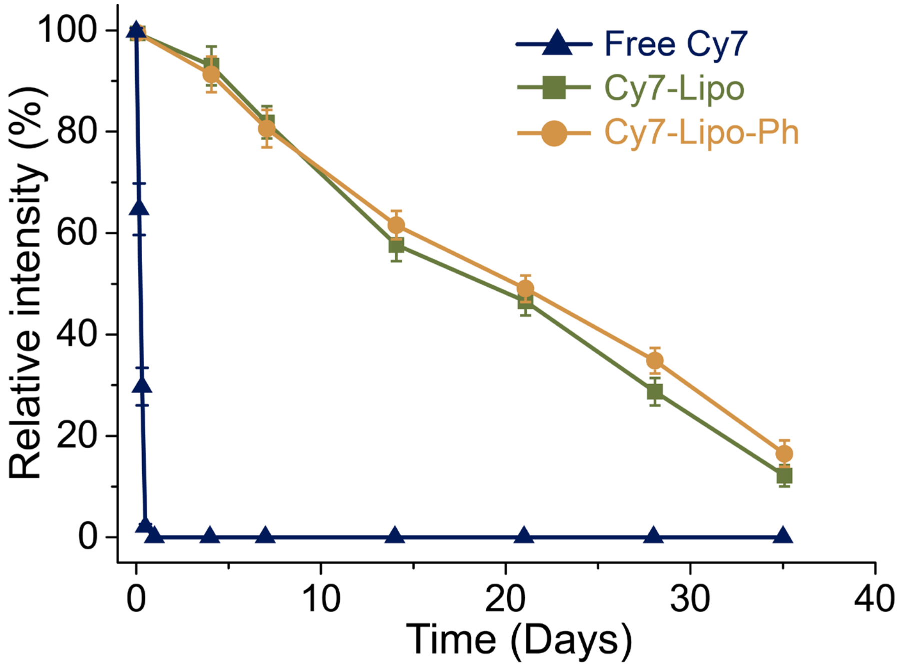

Supplementary Figure 16. Quantification of the fluorescence intensity over time (as a percentage of fluorescence at time=0, immediately after injection), derived from data such as those in Fig. 4a. Navy triangle, green square and orange circle represent free Cyanine 7 dye (Cy7), Cy7-conjugated liposomes (Cy7-Lipo) and Cy7-conjugted aromatized liposomes (Cy7-Lipo-Ph), respectively. Data are presented as mean ± SD, n =4 biologically independent animals.

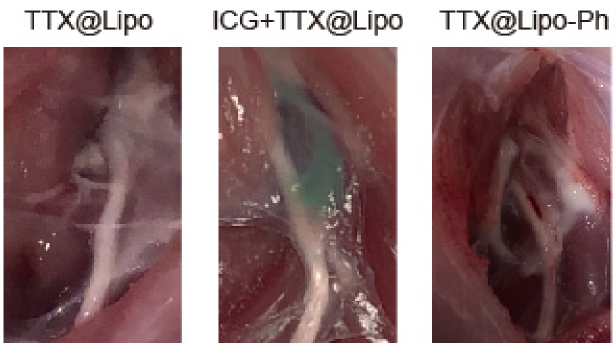

Supplementary Figure 17. Representative photographs of the site of injection upon dissection 14 days after injection of different liposome-TTX formulations. The green color is due to ICG.

Supplementary Tables

Supplementary Table 1. Effect of aromatization on the loading of different payloads. Data are presented as mean ± SD, n = 4 independent experiments. p-values are from unpaired two-tailed t-test. p-values compare unmodified liposomes and aromatized liposomes encapsulating different payloads. **P* < 0.05. ***P* < 0.01. ****P* < 0.001.

Payload	Drug loading			
	Lipo	Lipo-Ph	Increase in loading	P values
SRho	2.6 ± 0.3%	3.1 ± 0.2%	19.2%	*P* = 0.0088
TTX	0.11 ± 0.01%	0.13 ± 0.01%	25.5%	*P* = 0.0042
Bup	3.5 ± 0.7%	5.6 ± 0.8%	60.0%	*P* = 0.0056
Dox	0.8 ± 0.1	1.1 ± 0.1%	30.4%	*P* = 0.0040
SRho-PEG1k	0.78 ± 0.04	0.87 ± 0.02	11.5%	*P* = 0.0106
SRho-PEG10k	1.64 ± 0.13	1.71 ± 0.02	4.3%	*P* = 0.2875
FITC-Ab	3.63 ± 0.20	3.78 ± 0.16	4.0%	*P* = 0.2929

Supplementary Table 2. Effect of aromatization on the release of different payloads. Data are presented as mean ± SD, n = 4 independent experiments. p-values are from unpaired two-tailed t-test. p-values compare unmodified liposomes and aromatized liposomes encapsulating different payloads. **P* < 0.05. ***P* < 0.01. ****P* < 0.001.

Payload	Cumulative release in first 24h			
	Lipo	Lipo-Ph	Decrease in release	P values
SRho	12.2 ± 0.5%	5.9 ± 0.7%	51.6%	P < 0.0001
TTX	17.1 ± 0.3%	11.3 ± 0.4%	34.0%	P < 0.0001
Bup	70.7 ± 1.9%	37.0 ± 2.7%	47.7%	P < 0.0001
Dox	26.8 ± 1.1%	17.6 ± 1.0%	34.3%	P < 0.0001
SRho-PEG1k	28.2 ± 2.5%	19.6 ± 2.0%	30.5%	P = 0.0016
SRho-PEG10k	26.2 ± 2.3%	14.5 ± 2.1%	44.6%	P = 0.0003
FITC-Ab	14.2 ± 1.2%	6.3 ± 0.8%	55.6%	P < 0.0001

Supplementary Table 3. Characterization of liposomal formulations.

Formulations	Diameter (μm)	Zeta Potential (mV)	Polydispersity Index
Lipo	1.21 ± 0.19	-29.2 ± 2.3	0.192 ± 0.061
TTX@Lipo	1.26 ± 0.14	-26.5 ± 1.2	0.122 ± 0.037
ICG+TTX@Lipo	1.38 ± 0.31	-29.9 ± 2.5	0.125 ± 0.070
TTX@Lipo-Ph	1.14 ± 0.17	-27.7 ± 3.5	0.159 ± 0.046

Data are means ± s.d., n = 4 independent experiments.

Supplementary Table 4. Comparison of loading efficiency and loading in different TTX formulations.

Formulations	Payload	Loading Efficiency (%)	Drug Loading (%)
Lipo	-----	-------------	-------------
TTX@Lipo	TTX	22.6 ± 1.9	0.106 ± 0.009
ICG+TTX@Lipo	ICG	84.3 ± 3.8	0.524 ± 0.024
	TTX	27.1 ± 1.7	0.127 ± 0.008
TTX@Lipo-Ph	TTX	28.5 ± 2.0	0.133 ± 0.009

Data are means ± s.d., n = 4 independent experiments.

Supplementary Table 5. In vivo assessment of TTX formulations.

Formulation	TTX dose (μg)	Duration of nerve block (h)	Contralateral peak latency (s)
Free TTX	4.0	2.5 ± 0.6	12 ± 0.0
TTX@Lipo	20.4	19.9 ± 4.4	7.5 ± 1.0
ICG+TTX@Lipo	24.4	26.9 ± 5.5	6.7 ± 0.9
TTX@Lipo-Ph	25.3	36.9 ± 4.6	3.3 ± 0.4
	32.1	57.1 ± 11.6	4.2 ± 0.5
	37.5	64.5 ± 8.1	5.0 ± 0.4
	44.2	70.7 ± 9.8	7.5 ± 1.2

Data are means ± s.d., n ≥ 4 rats per group.

Supplementary Table 6. Inflammation 4 and 14 days after injection of formulations.

Liposomal formulation	Inflammation Scores			
	Day 4	P value	Day 14	P value
Untreated	0 (0-0)	-	0 (0-0)	-
Free TTX	0 (0-1)	0.8353	0 (0-0)	> 0.9999
TTX@Lipo	3 (3-3)	< 0.0001	2 (2-2)	0.0015
ICG+TTX@Lipo	2 (2-2)	< 0.0001	1 (0-2)	0.0164
TTX@Lipo-Ph	2.5 (2-3)	< 0.0001	2 (1-3)	0.0015

Inflammation scores: 0–4. Data are medians with 25th and 75th percentiles in parentheses; n = 4 biological independent animals per group. Statistical analysis was performed using one-way ANOVA with a Tukey post hoc test. P values result from the comparison of liposomal formulations to the untreated group at Day 4 or Day 14.

Supplementary Table 7. Myotoxicity 4 and 14 days after injection of formulations.

Liposomal formulation	Myotoxicity Scores			
	Day 4	P value	Day 14	P Value
Untreated	0 (0-0)	-	0 (0-0)	-
Free TTX	0 (0-0)	> 0.9999	0 (0-0)	> 0.9999
TTX@Lipo	0.5 (0-4)	0.5303	0.5 (0-1)	0.9383
ICG+TTX@Lipo	0 (0-0)	> 0.9999	0 (0-0)	> 0.9999
TTX@Lipo-Ph	0 (0-0)	> 0.9999	0.5 (0-4)	0.5665

Myotoxicity scores: 0–6. Data are medians with 25th and 75th percentiles in parentheses; n = 4 biological independent animals per group. Statistical analysis was performed using one-way ANOVA with a Tukey post hoc test. P values result from the comparison of liposomal formulations to the untreated group at Day 4 or Day 14.
