# Supplementary material for: Aromatized liposomes for sustained drug delivery
Source: Nat Commun. 2023 Oct 20;14:6659. doi: 10.1038/s41467-023-41946-8 (PMC10589217; doi:10.1038/s41467-023-41946-8)
Supplement: Supplementary file 2 — Reporting Summary [file 41467_2023_41946_MOESM2_ESM.pdf]

nature portfolio

Corresponding author(s): Daniel S. Kohane

Last updated by author(s): Sep 11, 2023

Reporting Summary

Nature Portfolio wishes to improve the reproducibility of the work that we publish. This form provides structure for consistency and transparency in reporting. For further information on Nature Portfolio policies, see our Editorial Policies and the Editorial Policy Checklist.

Statistics

For all statistical analyses, confirm that the following items are present in the figure legend, table legend, main text, or Methods section.

n/a	Confirmed	
☐	☒	The exact sample size (*n*) for each experimental group/condition, given as a discrete number and unit of measurement
☐	☒	A statement on whether measurements were taken from distinct samples or whether the same sample was measured repeatedly
☐	☒	The statistical test(s) used AND whether they are one- or two-sided<br>*Only common tests should be described solely by name; describe more complex techniques in the Methods section.*
☐	☒	A description of all covariates tested
☒	☐	A description of any assumptions or corrections, such as tests of normality and adjustment for multiple comparisons
☐	☒	A full description of the statistical parameters including central tendency (e.g. means) or other basic estimates (e.g. regression coefficient) AND variation (e.g. standard deviation) or associated estimates of uncertainty (e.g. confidence intervals)
☐	☒	For null hypothesis testing, the test statistic (e.g. *F*, *t*, *r*) with confidence intervals, effect sizes, degrees of freedom and *P* value noted<br>*Give P values as exact values whenever suitable.*
☒	☐	For Bayesian analysis, information on the choice of priors and Markov chain Monte Carlo settings
☒	☐	For hierarchical and complex designs, identification of the appropriate level for tests and full reporting of outcomes
☒	☐	Estimates of effect sizes (e.g. Cohen's *d*, Pearson's *r*), indicating how they were calculated

Our web collection on statistics for biologists contains articles on many of the points above.

Software and code

Policy information about availability of computer code

Data collection	No software was used.
Data analysis	Statistical analyses were performed using GraphPad Prism 8. Microsoft for Microsoft 365 (Version 2308). Graphpad Prism were used for data analysis. OriginPro 9.1 was used for plotting. Image analysis and scaling were then done through ImageJ (Fiji) software.

For manuscripts utilizing custom algorithms or software that are central to the research but not yet described in published literature, software must be made available to editors and reviewers. We strongly encourage code deposition in a community repository (e.g. GitHub). See the Nature Portfolio guidelines for submitting code & software for further information.

Data

Policy information about availability of data

All manuscripts must include a data availability statement. This statement should provide the following information, where applicable:

- Accession codes, unique identifiers, or web links for publicly available datasets
- A description of any restrictions on data availability
- For clinical datasets or third party data, please ensure that the statement adheres to our policy

The data that support the findings of this study are available within the article and its Supplementary Information files. The raw numbers for charts and graphs are available in the Source Data file whenever possible. Source data are provided with this paper. The full image dataset is available from the corresponding author upon request.

Research involving human participants, their data, or biological material

Policy information about studies with human participants or human data. See also policy information about sex, gender (identity/presentation), and sexual orientation and race, ethnicity and racism.

Reporting on sex and gender	N/A
Reporting on race, ethnicity, or other socially relevant groupings	N/A
Population characteristics	N/A
Recruitment	N/A
Ethics oversight	N/A

Note that full information on the approval of the study protocol must also be provided in the manuscript.

Field-specific reporting

Please select the one below that is the best fit for your research. If you are not sure, read the appropriate sections before making your selection.

☒ Life sciences ☐ Behavioural & social sciences ☐ Ecological, evolutionary & environmental sciences

For a reference copy of the document with all sections, see nature.com/documents/nr-reporting-summary-flat.pdf

Life sciences study design

All studies must disclose on these points even when the disclosure is negative.

Sample size	No statistical methods were used to predetermine size. Sample size were selected based on review of published literature where similar types of experiments were conducted.
Data exclusions	No data were excluded.
Replication	All the reported results were repeatable. Replicates were used in all experiments, as noted in the figure legends and in Methods.
Randomization	Animals were randomly assigned to one of the experimental groups. All samples/experiments were randomly allocated into experimental groups.
Blinding	All scoring and other histological assessments were performed by an observer blinded as to the nature of the individual samples. For all other experiments, the investigators were blinded during sample collections and subsequent measurement. The analysis was done objectively and without bias.

Reporting for specific materials, systems and methods

We require information from authors about some types of materials, experimental systems and methods used in many studies. Here, indicate whether each material, system or method listed is relevant to your study. If you are not sure if a list item applies to your research, read the appropriate section before selecting a response.

Materials & experimental systems

n/a	Involved in the study	
☒	☐	Antibodies
☐	☒	Eukaryotic cell lines
☒	☐	Palaeontology and archaeology
☐	☒	Animals and other organisms
☒	☐	Clinical data
☒	☐	Dual use research of concern
☒	☐	Plants

Methods

n/a	Involved in the study	
☒	☐	ChIP-seq
☒	☐	Flow cytometry
☒	☐	MRI-based neuroimaging

Eukaryotic cell lines

Policy information about cell lines and Sex and Gender in Research

Cell line source(s)	C2C12 mouse myoblasts (American Type Culture Collection (ATCC) CRL-1772) and PC12 rat adrenal gland pheochromocytoma cells (ATCC, CRL-1772).
Authentication	Prior to distribution, ATCC uses STR analysis to screen C2C12 and PC12 cell lines for authenticity and purity. Upon receipt of these cell lines and after plating, a morphology check by microscope on both C2C12 and PC12 is done to ensure authenticity.
Mycoplasma contamination	Cells were not tested in our laboratory for mycoplasma contamination.
Commonly misidentified lines (See ICLAC register)	No commonly misidentified cell lines were used.

Animals and other research organisms

Policy information about studies involving animals; ARRIVE guidelines recommended for reporting animal research, and Sex and Gender in Research

Laboratory animals	10-week old adult male Sprague–Dawley rats (Charles River Laboratories) weighing 350–400 g were used.
Wild animals	No wild animals were used in the current study.
Reporting on sex	Sex is not considered in the animal studies. We have shown that there is no difference in the duration of sustained release local anesthetics between male and female rats (Pharm. Res., 2019, 179).
Field-collected samples	The study did not involve samples collected from the field.
Ethics oversight	All procedures associated with animal studies followed recommendations in the Guide for the Care and Use of Laboratory Animals of the National Institute of Health. The Institutional Animal Care and Use Committee (IACUC) at Boston Children's Hospital approved the protocol (protocol 17-07-3497R).

Note that full information on the approval of the study protocol must also be provided in the manuscript.
